# Stakeholder engagement in European research and innovation: An investigation into how and why EU R&I projects develop engagement tools

**DOI:** 10.12688/openreseurope.19907.2

**Published:** 2025-09-09

**Authors:** Luka Gudek, Madhura Rao, Jacqueline Broerse

**Affiliations:** 1 Vrije Universiteit Amsterdam Athena Instituut, Amsterdam, North Holland, 1081 HV, The Netherlands

**Keywords:** EU, R&I, stakeholder engagement, tool, innovation, mission-oriented

## Abstract

**Background:**

The European Union’s research and innovation (R&I) efforts have increasingly prioritised collaboration, co-creation, and stakeholder engagement to address complex systemic challenges in recent decades. However, while stakeholder engagement has become a cornerstone of EU innovation policy in this area, there has been limited research into how tools supporting stakeholder engagement are developed, deployed, and sustained within R&I projects. To address this gap, this article explores factors influencing the development of stakeholder engagement tools in European R&I projects and their relation to the broader European R&I trends.

**Methods:**

This study adopts a qualitative approach, with conducting semi-structured interviews with 22 participants representing 14 Horizon Europe projects on topics of agri-food, bioeconomy, and sustainability. Data were collected, coded, and analysed concurrently and the emerging results guided which group was approached next.

**Results:**

Tools developed within projects take up shape within distinct phases, denominated as Purpose, Prototyping, Praxis, and Post-project continuity. Each of these phases comes with distinct challenges and opportunities. The way projects approach these challenges and opportunities showcases two distinct approaches that might be indicative of broader project management work in European R&I projects. These are the Project-focused approach and the User-focused approach.

**Conclusions:**

This study contributes to the broader discourse on innovation by providing empirical insights into the mechanisms and dynamics of stakeholder engagement tools in R&I projects. It underscores the importance of balancing structural R&I frameworks with flexible, participatory approaches to innovation. The findings offer actionable recommendations for policymakers, project coordinators, and funders to support the development of robust and inclusive stakeholder engagement tools that mobilise diverse actors and facilitate systemic change aligned with the EU’s sustainability goals.

## Plain Language Summary

Research funded by the European Union often takes place through collaborative projects in which universities, expert organisations, and businesses take part. These projects often develop tools such as games, guidelines, and exercises to bring the research they do closer to the public. The tools developed within projects can be used to do the research with the public, inform them of the results of the project, or provide a service.

Interestingly, the way in which these projects develop the tools for engaging with the public can take different trajectories. Some projects focus on transparency and accountability towards the EU, as the funder of their work. Other projects focus more on connecting with the public in every stage of developing the tool. We call these two different approaches the project-focused approach and user-focused approach. Each of these approaches can be applied more generally in European research and innovation.

This study argues that the trajectory of tool development in European projects is shaped both the rules set by the EU and the way that staff in the organisations participating in projects approach their work.

## Introduction

In recent decades, Europe ^1^ has grappled with several challenges, including geopolitical crises, the climate crisis and biodiversity loss, maintaining global competitiveness, and others. These grand challenges encompass both environmental and societal aspects and addressing them requires directing societal transitions through ambitious innovation policy ( Mazzucato, 2016). To achieve this, the European Union (EU) employs wide-ranging Research and Innovation (R&I) activities, increasingly turning to models of innovation such as Transformative Innovation Policy and Mission-oriented Innovation Policy which explicitly attempt to turn ambitions of resolving these challenges into actionable policy agendas ( Bergek *et al.*, 2023). However, given the elusive nature of societal transitions, the EU must adopt a distinctly novel and reflexive approach to R&I policy – one that not only acknowledges the complexity of existing system dynamics but also actively navigates and reshapes them to drive meaningful transformation ( Borrás & Edler, 2020; Mazzucato, 2016).

Additionally, R&I funders, such as the EU, increasingly recognise that innovation for these grand challenges must include expansive and dynamic constellations of actors working together ( Howoldt & Borrás, 2023). In recent years, the EC has therefore introduced various efforts to increase the collaboration and engagement aspects of its R&I policy, to enhance understanding of system dynamics, and to increase legitimacy and facilitate impact of R&I results ( Michali & Eleftherakis, 2022). These efforts have included increased co-creation and inclusion with society under the concept of Responsible Research and Innovation (RRI) ( Burget *et al.*, 2017) and Open Science ( Armeni *et al.*, 2021), and more recently a focus on aligning various societal actors around measurable innovation targets in Mission-Oriented Innovation Policy (MOIP) ( Robinson *et al.*, 2021). Prioritisation of these engagement instruments in R&I could support innovation efforts in various ways, including ensuring the relevance, longevity, and application of R&I processes ( Mazzucato, 2019). At the same time, research engaging with RRI tools points to a disparity between theory and practice, as well as a need for more empirical knowledge on implementation of RRI processes ( Marschalek *et al.*, 2017).

Engagement within European R&I processes takes many different forms. An interesting example is that of various engagement tools developed within R&I projects. Within the context of European R&I, the term 'engagement tool' encompasses a range of interpretations; however, for the purposes of this study we define it as actionable guidelines and practical processes on how to engage with various stakeholders on a specific topic in the context of R&I. Practically, these tools can take shape as scripts for multi-stakeholder workshops, digital data sharing platforms, or networking apps. While recent research has explored the overall success factors of European R&I projects (e.g. Tenhunen-Lunkka & Honkanen, 2024), no studies specifically focus on the tools developed and deployed within these projects, despite their importance for enhancing stakeholder engagement. Investigating this gap is particularly important as recent research points to the failures of the wide collection of engagement tools to support the implementation of RRI in Horizon 2020 ( Tabarés *et al.*, 2022).

Therefore, this study aims to explore factors that influence the development of stakeholder engagement tools in European R&I projects and their relation to the broader European R&I trends. To this end, the study tackles the question: How are engagement tools developed in R&I projects and what are the underlying logics of these processes? The paper proceeds with an overview of the development of the current European R&I system and the role of food systems R&I within it. This is followed by an empirical analysis of tool development processes within R&I projects focusing mostly, but not exclusively, on engagement in food system transformation. Finally, two distinct approaches to development of these tools are outlined.

## European R&I for food system transformation

Ever since the beginning of the European Community, Europe has had a role in coordinating and stimulating R&I activities. This role expanded from bounded technological research – for example on coal and nuclear energy – to the subsequent introduction of transnational cooperation in R&I and the use of Framework Programmes (FPs), which outline the scope and priorities of R&I while also serving as financial instruments ( European Parliament DG for Parliamentary Research Services, 2017).

The role of engagement in R&I has evolved significantly since the adoption of the first Framework Programme (FP). By the time FP6 was adopted in 2000, there was an established focus on the interaction between science, and society and citizens, including a thematic area on 'citizens and governance in a knowledge-based society' ( European Parliament DG for Parliamentary Research Services, 2017). Building on this, the Horizon 2020 programme (the precursor to Horizon Europe) introduced key elements of Responsible Research and Innovation (RRI), including “public engagement, policy deliberation, participatory research agenda-setting, and citizen science” ( Robinson *et al.*, 2021, p. 210). The current FP, Horizon Europe (2021 to 2027) aims to address climate change, achieve Sustainable Development Goals (SDGs), and enhance EU’s competitiveness ( European Commission - DG R&I, 2024b). It introduces specific instruments such as EU missions. These are a novel R&I instrument aimed at aligning innovation with systemic challenges faced by the EU through collaboration and engagement across various levels of governance and diverse societal stakeholders ( European Commission - DG R&I, 2023; Mazzucato, 2019). Additionally, European partnerships support bringing together private and public partners to work on grand challenges through R&I. While Horizon Europe presents innovative R&I approaches (e.g. explicit normative orientation and new methods for engagement with society), further policy focus on implementation of innovation policy with society is needed ( Haddad *et al.*, 2022; Kastrinos & Weber, 2020).

Even though Horizon Europe introduces various novel engagement instruments to European R&I, such as missions and partnerships, most R&I activities take place through a project-based approach. This is achieved by publishing calls for project proposals, aiming to build international consortia around a certain topic, with a specific scope and envisaged outcomes ( European Commission - REA, 2024). Within Horizon Europe, food systems are an important focus. Food systems bring together a wide array of actors, from small-scale farmers and food processors to policymakers, consumers, and multinational corporations and face a wide variety of urgent complex environmental, social and health challenges. In 2016, Food 2030 was launched as a “research and innovation policy framework supporting the transition towards sustainable, healthy and inclusive food systems, that respect planetary boundaries” ( European Commission - DG R&I, 2024c). This framework operationalises the challenges of the transition in the food system by outlining thematic priorities and specific pathways to achieving sustainable, healthy, and just food systems. This is achieved through innovation tools such as Partnerships and Flagship Missions that aim to engage broad societal groups for challenge-led innovation processes ( European Commission - DG R&I, 2024a; European Commission - DG R&I, 2024c). This highlights the complexity of the European R&I environment, while at the same time emphasizing the importance of engagement in R&I activities.

While engagement with various societal actors has become more prevalent in EU policy documents and academic literature, reflections on engagement are also explored in different scientific fields. The concept of stakeholder engagement gained salience in the 1980s in domains such as Business Organisation (cf. Freeman, 2010), Constructive Technology Assessment ( Rip, 2018), and Participatory Technology Development (within agricultural development domain). More recently, in 2010s, the increased incorporation of engagement approaches into EU R&I has been a result of the Responsible Research and Innovation concept, which builds on previous research on stakeholder engagement, emphasising inclusion, anticipation, responsiveness and reflexivity in innovation processes ( Burget *et al.*, 2017). Within the European R&I projects, the institutionalisation of RRItook place through the development of methods to engage with the public, as well as making those freely available ( Owen *et al.*, 2021). Within RRI, those to be engaged are often referred to as stakeholders (cf. Koch, 2020) to encompass the different groups and interests to be engaged. As the work on engaging various societal actors in policy grew in importance, it was also integrated into research within the sustainability transitions field, with engagement of stakeholders being emphasised as crucial for achievement of transformative objectives ( Huttunen *et al.*, 2022). 

The project-based work can be seen as a desirable format as it allows the EC to bring R&I closer to citizens as well as allowing to ‘manage the unamanageable’ challenges’ (cf. Tenhunen-Lunkka & Honkanen, 2024). However, the project logic of European R&I itself might structurally fence off certain actors, asserting specific manners of work, and reproducing employment uncertainty, thereby limiting the impact of engagement instruments (e.g. living labs) through *projectification* ( Büttner & Leopold, 2016; Erisman *et al.*, 2024). Recent studies indicate that achieving meaningful engagement and transformative ambitions of projects could be hampered by the wider project logic of Horizon Europe, which often leads to depoliticization and inability to address deep injustices ( Luger *et al.*, 2023). Furthermore, more critical perspectives on engagement in transformative R&I point out that mission-led approaches sometimes fail to resolve issues such as stakeholder monotony (cf. Kirchherr *et al.*, 2023), and lack of consideration for geographical diversity ( Uyarra *et al.*, 2025).

Failing to meaningfully engage with actors in these processes can have consequences such as stakeholder fatigue when stakeholders are engaged in extractive ways rather than seeking their input at all stages of projects ( Bracken *et al.*, 2015). Additionally, empirical research shows that bureaucratic and political resistance to participative processes regularly means that engagement does not translate to policy action ( Wesselink *et al.*, 2011). In R&I processes, the input of stakeholders is often not evaluated equally, with citizens’ voices frequently being undervalued. Furthermore, even when meaningful participation processes are designed, the political nature of missions might reinforce tensions and polarisation around certain societal issues ( Janssen *et al.*, 2021). This makes it important to gain insight into the process by which the projects implement engagement activities.

Many projects attempt to connect more directly with society through their outcomes. Recently, there has been a trend toward operationalizing these outcomes as ‘tools’ that can be used to engage stakeholders. These tools are designed to engage different users with the core themes of a project. However, their development processes, objectives, methods for reaching stakeholders, and post-project applications remain understudied. The development logics of these tools in R&I projects likely reflect generalisable approaches to tool development. For example, design thinking is deployed as an iterative and intentionally non-linear approach to identifying and solving problems in the private sector and academia and comprises of a range of methods ( Luchs, 2015). Such non-linear approaches attempt to create societally robust knowledge through inclusion of diverse stakeholder perspectives and types of knowledge ( Regeer *et al.*, 2009).

On the other hand, the context of EU R&I environment asserts restrictions and specific resource strains on organisations taking part in R&I processes (e.g. Lages *et al.*, 2023), which may limit what kind of approaches to tool development are feasible. This might encourage more linear-oriented tool development approaches. This study therefore delves deeper into the empirics of tools development within the EU R&I environment in order to understand what kind of logics govern their design.

## Methods

### Data collection

For this study, we conducted semi-structured interviews with 22 participants representing 14 Horizon Europe projects. Out of these, nine projects focus on food and agricultural systems, four on the bioeconomy, and one sector agnostic project. Between one and three project staff members were interviewed per project, depending on their level of involvement in the tool development process. If a participant had extensive knowledge of this process, no further interviews were conducted for that project. However, if an interviewee had only partial knowledge, additional participants were interviewed to ensure a comprehensive account of the development process. The study focuses on a subset of Horizon Europe projects, namely the projects under the Food 2030 umbrella, attempting to address the challenges of food system transformation. Potential projects were identified via a desk study involving the analysis of all ongoing and recently concluded Horizon Europe food and agriculture projects. This thematic subset of projects is a result of the authors’ work on the CLEVERFOOD project which engages broadly with food system related projects. Projects websites were scanned for engagement tools. Subsequently, coordinators of all projects that had developed or were in the process of developing such tools were contacted by the first and the second authors. Table 1 presents an overview of the included projects, the tools developed by them, as well as which stakeholders they tried to engage. As the projects attempt to engage diverse societal actors, such as policy makers, food producers, researchers, businesses, we refer to these collectively as stakeholders taking part in stakeholder engagement processes. When appropriate, we refer to specific stakeholder groups. Initially, interviews were conducted with representatives of 17 projects, but three projects were excluded from the analysis due to the tools not being sufficiently focused on stakeholder engagement. The full interview guide is available and listed in references ( Gudek *et al.*, 2025a).

**Table 1.** Overview of included projects and tools.

Project	Engagement tool(s)
*Agri-food projects*	
AGROBRIDGES	*Net*: An online platform that brings together local agri-food actors to cooperate and share resources and infrastructure to create stronger short food supply chains. *Smart delivery*: An online space to create local delivery systems of agri-food products using the short food supply chain model. *Hear my voice*: An online space that brings together farmers, producers, consumers and other agri- food actors to present ideas for new short food supply chains and find supporters and partners to bring their ideas to life. Targeted stakeholders: Local food producers and farmers, Retailers, Food cooperatives, Local policy makers, Citizens.
CULTiVATE	*The Library of Citizen Engagement* **:** An open-access collection of strategies and resources designed to enhance public participation in food sharing initiatives. It provides a curated set of best practices, case studies, and practical guidelines to help organisations foster community involvement, scale up their activities, and build more inclusive, sustainable food-sharing networks. *Community of Practice Amplification Programme* **:** A structured knowledge exchange initiative aimed at strengthening collaboration among food sharing initiatives across different regions in Europe. It connects stakeholders through workshops, mentoring, and peer-learning sessions, enabling them to share experiences, refine engagement strategies, and co-develop innovative solutions for more effective and resilient food-sharing ecosystems. Targeted stakeholders: Researchers, Policy makers, Food sharing initiatives, Citizens.
ECO-READY	*Real-Time Surveillance System (Observatory)*: A digital platform providing real-time assessments and frequent updates on the food system, accessible via web and mobile applications for policymakers, scientists, the agri-food industry, and the public. *Early Warning System and Decision Support Tools*: AI-driven prediction models offering timely alerts and guidance to stakeholders, helping them respond proactively to emerging food security challenges. *Policy Hub:* A platform providing continuous policy advice to address shocks and contingencies affecting food security, ensuring that future policies align with the needs of European farmers and society. Targeted stakeholders: Researchers, Policy makers, Agri-food industry, Citizens.
FIT4FOOD2030	*Tools for transformation*: The toolkit offers over 60 tools and resources to support food system transformation. The tools have a strong focus on co-creation, shared learning, and collaboration amongst a wide range of stakeholder across the food system. It includes process-oriented tools for setting up innovation labs and R&I policies, as well as awareness-raising tools to explore food system challenges. Targeted stakeholders: Researchers, Facilitators, Food specialists, Business representatives, Policy makers, Civil society, Citizens.
FOODITY	*FOODITY datalake*: A centralised platform for collecting, organising, and sharing diverse food and nutrition data, including nutritional values, recipes, and emerging food trends. It provides businesses, researchers, and food enthusiasts with structured and reliable datasets to drive innovation in the food sector. The platform ensures data security while facilitating collaboration across culinary and food research communities. Targeted stakeholders: Researchers, Food policy experts, Small and medium enterprises, Civil society
FoodSafety4EU	*Expert Finder App*: A networking tool that helps professionals in the food safety sector connect, share knowledge, and build collaborations across institutions, authorities, industries, SMEs, and associations. *Project Finder App*: Enables users to discover ongoing and completed food safety projects, fostering knowledge exchange and collaboration among researchers and industry stakeholders. Targeted stakeholders: Researchers, Policy makers, Project coordinators, Industry representatives, Citizens.
FoodShift	*Innovation Cases*: A collection of around 80 food system innovations from across Europe, categorised by city and innovation type (product, process, social, governance). These serve as practical examples and inspiration for cities and stakeholders looking to implement similar initiatives. *Knowledge Base*: A repository of background materials, methodologies, and best practices covering Governance, Sustainability, Job Creation, and Citizen Empowerment. This section provides guidance on improving local food system governance, enhancing sustainability, creating employment opportunities, and engaging communities. *City Stories*: Narratives from nine FoodSHIFT2030 city regions and additional fellow cities, showcasing unique approaches to food system transformation. These stories offer valuable insights, lessons learned, and strategies that can inform and inspire other cities on their transition journeys. Targeted stakeholders: Researchers, City officials, Food businesses, Citizens.
FOX	*FOXLINK App: A* communication platform developed to enhance interactions among consumers, local food producers, and food scientists across six European regions. Its primary goal is to facilitate the exchange of information and feedback, thereby strengthening local food networks and promoting sustainable practices. Targeted stakeholders: Food scientists, Local food producers, Consumers.
FUSILLI	*Urban Food Knowledge Community Platform (KCP)*: A knowledge-sharing and learning platform compiling lessons learned, case studies, and best practices from various regional and urban food systems across Europe. It provides resources such as transformation journeys, project reports, and showcases to guide stakeholders in sustainable food system practices. Targeted stakeholders: Living lab coordinators.
*Bioeconomy projects*	
BlueRev	*BlueRev Support Tool*: An e-learning platform to support the revitalisation of the blue bioeconomy. It offers training courses, webinars, an e-library, and a community feed for knowledge-sharing and networking among stakeholders. The platform is designed to foster cross-sector collaboration, particularly in Denmark, Italy, and Estonia, by providing tailored resources and interactive learning opportunities. Targeted stakeholders: Policy makers, Company managers, Blue economy specialists.
ALLthings. BioPRO	*Mission BioHero*: A serious game featuring interactive campaigns covering themes such as food packaging, fashion and textiles, schools, and careers in the bioeconomy. Players complete quizzes, real-life tasks, and mini-games to transform a polluted city into a sustainable one, while learning about the bioeconomy in an engaging way. *Label BioHero*: A mobile application that allows users to scan eco-labels and certifications on bio-based or biodegradable products. It provides detailed sustainability information, helping consumers make informed choices and increasing awareness of bio-based alternatives. Targeted stakeholders: Citizens.
FER-PLAY	*FER-PLAY database*: An online database cataloguing alternative fertiliser value chains derived from various types of biological raw materials. Stakeholders (farmers, companies, regulators) can explore this tool to understand the characteristics, benefits, and performance of various circular fertilisers. By visualising life-cycle data and case studies, the platform supports evidence-based decision-making and knowledge sharing across regions. Targeted stakeholders: Business decision-makers and policy makers.
UNaLAB	*UNaLab Toolkit*: A collection of co-creation tools designed to support the development, implementation, and evaluation of nature-based solutions in urban areas. It is structured into five categories: Need Finding, Ideation, Strategy, Experimentation, and Feedback, guiding users through the innovation process. The toolkit includes games, workshops, templates, and ICT tools to facilitate stakeholder collaboration and citizen engagement. These resources help cities and organisations co- create sustainable solutions tailored to local needs. Targeted stakeholders: Living lab coordinators.
Sector-agnostic project	
RRI tools	*RRI Tools:* A toolkit created with the aim of promoting research and innovation aligned with societal values and needs. This online repository offers a wealth of resources, including knowledge products, practical tools, inspiring practices, and project examples, all designed to guide stakeholders in implementing RRI principles across various contexts. The toolkit is tailored for a diverse audience— such as researchers, policymakers, educators, industry professionals, and civil society organizations— and facilitates the integration of RRI into their practices. Users can filter resources based on criteria like stakeholder group, topic, required expertise, related social challenges, and language, making it a versatile and user-friendly platform for advancing responsible research and innovation. Targeted stakeholders: Researchers, Policy makers, Civil Society, Educators.

We selected interviewees through a theoretical sampling strategy ( Glaser & Strauss, 2017). As per Glaser and Strauss, while applying this strategy, the process of data collection is controlled by the emerging theory. The initial decisions pertaining to data collection are based on the general problem area and not on a preconceived theoretical framework. Data were collected, coded, and analysed concurrently and the emerging results guided which group or sub-group to turn to next ( Glaser & Strauss, 2017). As a result of this, although we started with projects with a strong focus on agri-food systems, midway through the data collection process, we decided to reach out to, and interview participants engaged in five other projects – four projects that had developed engagement tools focused on the bioeconomy and one sector-agnostic project focused on stakeholder engagement in research and innovation in general. By including participants who worked on engagement tools outside the food systems context, we aimed to understand whether the divergence in their focus might reveal alternative perspectives or challenge the assumptions we had developed. This approach was intended to uncover any inconsistencies or unique factors that might not have been evident within the food systems projects alone, thereby strengthening the robustness of our findings. Ultimately, none of the interviewees pointed to contrary experiences in non-food projects they had coordinated. Nevertheless, the strong representation of agri-food projects suggests that the findings might not be fully generalisable to all thematic areas of Horizon Europe R&I landscape and may need to be further validated.

The study participants were informed in advance about the purpose of the study and were promised anonymity. All interviews lasted around 60 minutes and were conducted online in English. The interview guide used by the authors has been included in Appendix 2. Excerpts of the interviews are used in the results and quoted verbatim, except when adjusted to improve readability. Descriptions of interviewees have been deliberately withheld to ensure their anonymity. Written and verbal informed consent to record the interviews, analyse the resulting data, and publish the findings, including anonymised excerpts, was requested and obtained from all participants.

### Data analysis

Qualitative data analysis software Atlas.ti was used for analysing the interview transcripts. Atlas.ti is proprietary qualitative data analysis software, which the authors accessed through the licence of their institution. Open-source alternatives to Atlas.ti are available, for example, QualCoder. The first step of our analysis was open coding ( Saldaña, 2021). The first and second authors undertook independent, line-by-line analysis of the text and assigned codes describing insights identified in the data. This was an iterative process and involved constant comparison between various pieces of text within the same transcript as well as with text in other interview transcripts. The researchers referred to the video recordings of the interviews from time to time during this stage of analysis to ensure that data were coded in relation to the contexts in which they were shared. Data saturation was reached at 10 interviews for the first author and 11 interviews for the second author, indicating that no new codes could be assigned to the data. As our analytical focus was on recurring patterns in how the tool development process was structured, participants’ motivations, and responses to key challenges, saturation was assessed in relation to these themes rather than across sectoral or technological dimensions. After this, the first and second authors compared their codes and combined similar codes. Dissimilar codes were discussed, and most were eventually integrated into the analysis. This resulted in 46 codes assigned to 257 pieces of text. The full coding tree is available and listed in references ( Gudek *et al.*, 2025b).

Subsequent to open coding, we engaged in a second cycle of analysis to group these codes into broader conceptual categories. Drawing on principles of axial coding ( Saldaña, 2021), we examined relationships among the codes, explored underlying patterns, and grouped them into higher-order categories. Through this process, it became evident that participants consistently described their activities in chronological sequences, structuring their accounts around distinct stages of tool development and dissemination. Based on this, we identified four phases of the tool development and dissemination process, as described in the consequent section of this paper. The 46 codes that were created during the previous analysis step were then assigned to one or more of the stages.

## Results

This section describes the four stages of the engagement tool development and dissemination process, as identified from the data: (1) purpose, (2) prototyping, (3) praxis and (4) post-project continuity. It illustrates the tasks undertaken by project stakeholders in every stage as well as the challenges they faced and strategies they employed to overcome them. Table 2 at the end of this section provides an overview of the results.

**Table 2.** Overview of the four phases of tool development.

	Identified challenges	Strategies (proposed or applied) to overcome identified challenges
Purpose	-Engagement tools developed by projects are often not genuinely relevant to the target group. -Stakeholders are hesitant to take part in development of tools when benefits are not yet tangible.	-Including potential users of tools as early as the conceptualisation stage into a co-creative process. -Conducting detailed stakeholder mapping and outlining their needs.
*This stage refers to the* * conceptualisation of engagement* * tools and understanding their target * *group.*		
Prototyping	-The input of stakeholders is valued unequally, with citizens often perceived as less knowledgeable. -Different jargon and communication styles used by technical and academic partners, and citizens create friction and delays in tool creation. -Pressure to achieve unrealistic goals. -Certain stakeholders have more power to choose how and when they take part in stakeholder engagement processes.	-Creating tailored engagement formats that fit the type of input sought by each group. -Redesigning the proposal evaluation procedures to avoid unrealistic expectations with regard to outputs and results.
*The prototyping stage is about the* * development of the tools as well as* * the negotiations with stakeholders* * and funding requirements.*		
Praxis	-Limited timeframes of projects make it difficult to monitor and evaluate the impact of the developed tools. -Dissemination of the tools often takes place towards the end of the project and with a limited budget. -Maintenance of tools, especially multilingual and translated tools, is costly and often results in only the English-language tools being maintained.	-Outlining monitoring and evaluation frameworks for tools at an earlier stage of the development process. -Dissemination and implementation plans should be outlined at the beginning of the project and sufficient time and budget should be allocated to connected activities.
*The praxis refers to the stage where* * the tools are publicised, translated* * and disseminated, and their impact* * monitored.*		
Post-project continuity	-No incentive to keep the tools active and up to date after the project ends. -Most projects create new tools instead of building on existing ones, which leads to duplication and multiple versions of the same tools.	-Designing follow-up projects and incorporating previously created outputs into them. -Additionally, obtaining external public and private funding. -Co-developing tools with ‘sister projects’.
*Post-project stage is about ensuring* * the tools remain available and* * maintained after the project that * *developed them runs out.*		

### Purpose: Assessing the need for engagement tools

The first step towards developing a stakeholder engagement tool entailed reflecting on the purpose of such a tool, the form it would take, and the stakeholders it would serve. While a general idea of a tool might be developed before the project as a part of the proposal, the critical conceptualisation of the tool happens at the beginning of the project. This subsection describes how participants define stakeholder engagement tools, the various approaches they take towards their conceptualisation, and the increasing focus in EU-funded food projects on considering the needs of marginalised stakeholders while developing engagement tools.

Interviewees indicated that their consortia developed stakeholder engagement tools to operationalise the knowledge created during the project and better involve relevant actors in achieving the project’s goals. Broadly, the aim was to create a tangible, well-structured output that could be used by stakeholders to undertake action to improve food systems. While this overarching aim appeared to be a shared one, interviewees expressed diverging views regarding what constituted a stakeholder engagement tool.

Some believed that " *anything can be a tool*" – an inspirational video, a workshop script, a policy brief, a set of best practices, a digital library, or a training programme for setting up open-innovation ecosystems such as Living Labs. These interviewees emphasised the flexibility and broad applicability of a tool in engaging stakeholders. They argued that tools should not be limited to conventional formats but should encompass various media and methods that inspire and educate stakeholders. Others were more cautious in their imagination of a tool and believed that a tool was something that could be ‘used’ by stakeholders as opposed to something that one could merely gain knowledge from. The projects of these interviewees developed tools such as games, mobile applications, databases, and decision-support tools. These tools were designed to be interactive and practicable, providing stakeholders with concrete resources to support their decision-making processes and actions. This view emphasised the importance of usability and functionality in tool development, ensuring that the tools have a direct and tangible impact on stakeholders' involvement in food systems transformation. Both these views could be considered top-down approaches to tool development wherein project partners determined which formats suited their goals best. In these cases, the expertise and vision of the consortium played a significant role in shaping the tools, leveraging their knowledge to create effective engagement strategies.

A few interviewees took a more user-centric approach wherein the needs of potential tool users were assessed first, and tools were developed accordingly. Discussing their perspective, a participant engaged in the Eco-Ready project shared:

“ *A tool needs to have well-defined users. When we talk about developing a tool, we need to ask who the potential users are and what benefits they will gain. A tool can be a methodology, a report, a set of policy recommendations, a contingency plan, or a roadmap that guides the user in making better-informed decisions. We can have a vast spectrum of tools in terms of format and complexity, as long as there is a potential user group that benefits from utilising the deliverable.*”

This approach prioritises the needs and preferences of the stakeholders, ensuring that the tools are relevant and beneficial to the intended users. By conducting baseline studies, stakeholder mapping exercises, and surveys, many projects aimed to assess the needs of various stakeholders, their interest in the project's topic, and their connectedness to other stakeholders in the food system. The outcomes of such exercises, along with project goals, determined what kind of tools were to be developed and why. The availability of resources and project partners' skills also influenced the feasibility and scope of tool development, guiding the selection of appropriate formats and methods to achieve the desired outcomes.

Better involving marginalised or overlooked actors in their projects was also an important aim for many interviewees when developing an engagement tool. Some were keen on involving groups that are not traditionally considered to be stakeholders in the food system. For instance, one project focused strongly on children and young people because its consortium believed that the new generation is keenly interested in the sustainability movement but frequently overlooked. Other projects focused on specific aspects of engagement, such as improving farmers' digital connectivity or enhancing the skills or knowledge of multiple stakeholder groups from a particular region on a specific topic. The inclusion of migrant workers and consumers with lower socio-economic position by creating tools in languages understood by them is another example of projects' efforts to include marginalised groups. Participants shared that a noteworthy challenge to including such stakeholders was convincing them that their opinion was relevant and needed for the tool prototyping. At an early stage, project partners often found it difficult to promise tangible outcomes and therefore struggled to convince marginalised groups regarding what they might gain from participating in the tool prototyping. Some projects addressed this challenge by offering the participants compensation in the form of food products for their time. This incentive has proven effective in engaging underrepresented participants by providing immediate and tangible benefits for their involvement. However, this is not the case for most projects, and they continue to struggle to involve marginalised participants despite recognising it as a critical area for improvement.

### Prototyping: Developing engagement tools in line with project aims and stakeholder needs

Once the purpose of developing a stakeholder engagement tool was established and there is an agreement about the form such a tool would take, the second stage – the prototyping of the tool – takes place. This subsection sheds light on characteristics of this stage such as frequent interaction with target user groups, increased cooperation between project partners, and bottlenecks connected to users as well as some project partners’ lack of familiarity with technology. Additionally, we will describe the frequently reported disconnect between the project proposal and the ground reality of tool development as described by interviewees.

Most participants described the tool prototyping stage as iterative and co-creative. Initially, project partners responsible for the tools convened to develop a prototype. Once such a version was ready, the tools were piloted with members of the target user groups, often in the form of co-creative workshops. Such pilot sessions and workshops facilitated dialogue between tool developers and potential users – be it facilitators or implementers – integrating practical insights with subject expertise. Interviewees emphasised the importance of involving end-users from the outset to ensure the tools were functional and user-friendly. However, different user groups’ opinions appeared to be evaluated differently. Interviewees sometimes exhibited bias towards the validity of the feedback that came from specialist groups such as policymakers, researchers and food systems professionals. On the other hand, user groups such as citizens were perceived as less knowledgeable, particularly about the feasibility of implementing ideas for technical tools like apps and websites that facilitated collaboration. A participant working on the AllThings.Bio project described their experience with citizens as follows:

“ *If you ask citizens for their opinion and let them be creative, they come up with all kinds of crazy ideas. But then you always have the developer with technical expertise who needs to see what can be done with the capacity and money we have*.”

The observation that power inequalities have direct consequences on which stakeholders get to engage in co-production in European R&I projects is increasingly highlighted in recent research (e.g. Cronin *et al.*, 2024). It was also noted that certain groups were better represented at co-creative sessions compared to others. Which groups these were depended on the project partners’ professional networks and the region where the sessions were held. However, stakeholders with steady employment and a personal interest in food systems transformation were found to be more represented in co-creative processes. In contrast, both the least powerful and the most powerful stakeholders were found to be harder to reach, because of lack of interest. An interviewee shared an example of this from a pilot session with potential end users of their tool:

“ *They [project partners] have really struggled to reach people who suffer from food insecurity because these people do not have the time nor enough faith in the system to go to such events. Then you also have stakeholders that are really powerful actors such as high earning farmers or supermarket executives and they are notoriously bad at attending any kind of co-creation events because they don’t see the benefit in it for them.*”

This often led to the same kind of stakeholders, or at times the exact same set of people, attending feedback or co-creative sessions, causing their preferences to be taken into account more prominently when refining engagement tools.

Interviewees described the tool development phase as requiring frequent cooperation and clear communication among project partners, which posed several challenges. When it came to developing tools based on digital platforms, partners with IT expertise such as coders and web developers often had different views on the feasibility of implementing user feedback compared to project coordinators, or research partners, often leading to discord. Additionally, academic partners were often described as “ *speaking a different language*” compared to other partners. These communication challenges sometimes resulted in project partners working in silos, contrary to the inter- and transdisciplinary nature of the project.

Accessibility was a key consideration in developing engagement tools, most of which were digital. Users’ affinity for digital technology often became central to the prototyping. Due to Europe’s well-developed digital infrastructure, the high ambitions of many projects, and increased move to digital work from the COVID-19 pandemic, many projects aimed to develop advanced digital tools. While these were well-received by some user groups, such as young people and educators, others were less keen on online engagement. An interviewee engaged in the AgroBridges project shared an example of this:

“ *We had one tool that was really not accepted by farmers – an online matchmaking tool. When we tested it, it was almost unanimously rejected by all farmers in all regions. They were annoyed by having to make their profiles, book meetings, and so on. They considered it very complex.*”

Besides some users’ lack of familiarity with digital technology, some project partners were also reported to lack the capacity to carry out tasks like updating a website or application. Consequently, partners with a better grasp of such processes had to take on these tasks, leading to an additional burden for them. Some interviewees shared that such challenges were the result of dissonance between what was promised in the project proposal and what was feasible considering time and resource restrictions. Those responsible for executing the project were often not involved in writing the project proposal and felt taxed by the pressure to deliver what was promised. An interviewee shared their thoughts about this as follows:

*“The project proposal was already written by my supervisors when I was hired. In the proposals, you can be as creative as you want. But when it comes to putting the proposed ideas into action, it's much more complicated*.”

The intense competition to acquire funding was described as a possible reason for overpromising. Further elaborating on how this was a systemic issue in EU-funded projects, they shared:

“ *I don't mean to sound arrogant, but the challenges we have faced in developing the tool have nothing to do with my own capacity or the capacity of the partners. It has everything to do with the fact that we're working on an EU-funded project and people who work on these projects are always structurally lacking time and resources to work together. And because of this, collaboration between the different partners has been really, really difficult*.”

### Praxis: Implementing and disseminating developed tools

Once the prototype for tools has been developed, participants reported an increased focus on piloting and implementing the engagement tools. In this subsection, we describe challenges such as the pressure to popularise their tools within a short period of time, managing translation, and evaluate the success and impact of the developed tools.

The implementation and dissemination of engagement tools involves several strategic steps designed to maximise their impact and ensure that they are effectively utilised by target stakeholders. As per interviewees, these steps often include publicising the tools at in-person events, leveraging their social media networks, enlisting the help of influential intermediaries, translating tool content to improve usability, and making adjustments to accommodate regional differences.

Most interviewees shared that they reached or expected to reach the implementation stage only towards the end of their projects. As a result, they faced considerable pressure to organise publicity campaigns and popularise their tools within a short period of time. For tools developed for professionals, LinkedIn was the most frequently used social media platform. For tools where citizens were seen as the target users, platforms like Instagram and TikTok were used. Although this had not yet been implemented for any of the projects in this study’s sample, some interviewees discussed the possibility of recruiting social media influencers to better publicise developed tools. In offline contexts, interviewees discussed popularising their tools via actors who could be considered ‘multipliers’. These could be consultants, advisors, educators, or anyone with a large network in the food industry. However, the budget for such endeavours was often limited. Regarding this, an interviewee shared:

“ *For a communication partner, it is very frustrating when there are so many excellent outputs that we would like to disseminate, but there's no money for dissemination. There is no money for infographics, social media campaigns, publicity events, or anything else.*”

A considerable amount of time and resources also need to be spent on translation at this stage. Many interviewees, especially those working on projects spanning several countries, shared that the effort required to translate tools was more than they had expected. Once the translation was underway or completed, making adjustments to the tools (whether session outlines, or digital platforms) also became a major challenge. Often, changes could be made to the original tool, which was usually in English, but there was a lack of resources to implement the changes in translated versions. Stakeholders from countries such as the Netherlands, Belgium and Finland, where English is not the native language but is widely spoken, were also found to be at an advantage since they could use original versions of the tools. However, most interviewees accepted translation and its management as an inherent part of European projects and saw intercultural collaboration as an opportunity to exchange knowledge and learn from different communities. Regarding this, one interviewee shared:

“ *This is the beauty of working in European projects, right? You get to bring together and learn from people from very different geographies, cultural backgrounds, contexts, food and farming practices, et cetera*.”

The implementation stage was also seen as a conducive period to evaluate the success and impact of the developed tools. Many interviewees monitored website visits and downloads as a way to measure success. For digital tools that required users to create accounts, it was possible to collect additional data about tool use. However, signing up or creating an account was also mentioned as a barrier to a tool’s uptake and therefore many interviewees preferred not to have it as a requirement. When asked about how they assessed the impact of their engagement tools, all interviewees shared that they did not think it was possible to do this within the timeframe of the project. They admitted that the metrics they assessed merely indicated the success of their publicity campaigns and were not indicative of a tool’s actual use or impact. Even over a longer period, assessing systemic change was thought of as difficult and at times, impossible to achieve. Additionally, since the projects often came to an end soon after the implementation stage, interviewees thought that there was no incentive to evaluate the success or impact of engagement tools. Reflecting on this, an interviewee shared:

“ *For some reason, the way we've organised research and innovation in Europe, only short-term accountability is emphasised. During the project, we have to write these huge reports for the European Commission where we indicate how impactful the projects have been. But once the project is over, we stop measuring its impact and we stop drawing on its value*.”

### Post-project continuity: What happens once funding runs out?

The last stage of the tool development and disseminations process entails ensuring the continued functioning and relevance of the tools. This subsection describes the various strategies such as the development of new related projects, seeking out private and public investors, and turning tools into marketable services that projects undertake to prolong the usability of their tools.

In most cases, the development, maintenance and promotion of engagement tools were feasible only while project funding persisted. This issue was frequently mentioned by interviewees spontaneously, but they were also specifically asked to comment on the future of the tools after their respective projects concluded. Having invested several years and significant resources into the tools, all interviewees concurred that these tools should remain available for public use for several years post-project. While digital tools like apps and websites are vulnerable to becoming unusable due to lack of maintenance, even methodologies and session outlines for workshops can become inaccessible if their hosting expires or external links in PDFs break over time. Typically, one of the project partners assumes the responsibility of maintaining the project website, where tools may be accessed for three to five years after the project’s completion. However, this usually entailed covering website subscription fees and little more. Other maintenance activities, such as updating a tool or responding to user queries, require additional resources and were deemed unfeasible without further funding. Often, the burden of maintaining tools fell on the project coordinators and their institutions. However, the limited resources and capacity within these organisations made it challenging to ensure continued functionality and relevance of the tools. Commenting on the pressure of such expectations, an interviewee shared:

“ *Post-project usability and helping new projects use existing tools are often seen as afterthoughts. Despite being very important pieces of the project, they're managed on a shoestring budget and on the goodwill of project partners. Often, this responsibility is pushed onto the project coordinator. However, it should also be the responsibility of the funders. Funding organisations need to seriously consider how the work that they have funded can be sustained in the long term and lead to transformative change*.”

One proposed solution to this dilemma was the establishment of follow-up funding mechanisms specifically targeting the sustainability of promising tools. Such mechanisms could involve a dedicated fund to support the continuation and refinement of tools deemed valuable. This would prevent these tools from falling into a " *project graveyard*" where they are no longer actively maintained or utilised. For example, the CLEVERFOOD project originated from the earlier FIT4FOOD project, serving as a continuation mechanism to expand and consolidate engagement tools. In the case of AgroBridges, the consortium was actively developing a new project proposal to build upon the tools they had created. The FER-PLAY team also reported that they were exploring opportunities to capitalize on the outcomes of their project by applying for new funding that would allow continued development of their tools.

Additionally, incorporating long-term planning into the initial project proposal could help address these challenges by allocating resources for post-project maintenance and updates from the outset. For example, when tools developed within a project are relevant to them, external public or civil society stakeholders such as national agencies, ministries, municipalities, and NGOs take over the tools and invest in their maintenance. This strategy often entails modifying tools to match the context in which these stakeholders wish to use them. However, this ensures that the tools remain free to access and useful to the public. In the FOX project for instance, a regional partner engaged with their local municipality to explore the possibility of continuing the use of a mobile unit and related app created as part of the project. Similarly, in ECO-READY, the project team discussed the possibility of leveraging strategic partnerships, including with the European Commission’s Joint Research Centre, to embed their tools into external platforms, while also using Living Labs to explore future sustainability options.

Another suggestion coming from the interviewees was to invest project resources of new projects to improve and build upon existing tools (and toolkits) instead of creating new products from scratch. Regarding this, an interviewee engaged in the FOX project reflected:

“ *There are so many great tools, toolkits, apps, and websites out there. Instead of enhancing these existing resources, we are wasting money creating something new just to have our project logo on it. It doesn't matter if the project logo is there, as long as the tool is useful and has a real impact.*”

Improved cooperation with other EU-funded projects with adjacent goals (often referred to as ‘sister projects’) and co-development of tools was suggested as yet another way to address the lack of sustainability currently experienced by projects. This could allow projects to save on resources in the tool prototyping in order to reserve funds for tool maintenance once the projects have concluded. Another strategy to build a new project around the developed tools. This strategy involves leveraging promising tools and expertise within the consortium to write proposals to acquire funding for new projects. By doing this, project partners ensured that their tools continued to evolve and remained relevant. This was the most popular continuation strategy discussed by interviewees. The CLEVERFOOD project, which seeks to develop an engagement toolkit, was frequently mentioned as a viable solution to prolong the life of the developed tools by establishing a toolkit hosting tools already developed by other projects.

Finally, some projects pursued potential commercialisation opportunities by transforming tools into marketable products or services; which could then be sold by a project partner. This approach requires developing a business model that can sustain the tool financially. Projects in this study explored various revenue streams, such as subscription models, licensing fees, or consultancy services, to support the tools’ maintenance and development. Commercialisation not only ensures the tool's survival but also its continuous improvement and adaptation to market needs. Some projects explored hybrid approaches, combining elements from all three categories to maximise their chances of sustainability. For example, they might integrate their tool into new projects while simultaneously seeking partnerships and exploring commercial opportunities. This approach also requires a critical perspective to a tool development to create a tool perceived as useful by potential users.

Overall, the insights shared by the participants allowed us to identify best practices and challenges associated with all phases of the tool development process. Table 2 presents an overview of this.

## Discussion

This study aimed to explore the factors influencing the development of stakeholder engagement tools in European R&I projects and their relation to the broader European R&I trends. As presented in the results section, tools for engagement developed as a part of European R&I projects support social innovation efforts of various projects. The development of these tools can be observed in distinct phases, relating to defining their purpose, the process of developing or designing them, implementing them in praxis, and their post-project continuity. This phased analysis of the tool development process allowed for a more structured examination of the different approaches that projects take to tool development. In addition, the results highlight the growing importance of a distinct approach centering end-users in the development process of stakeholder engagement tools. After discussing the two approaches to tool development, and a need to transition to socially robust tools, this section ends with a reflection on the limitations of the study.

### Project-focused and user-focused approaches to tool development

Based on the tool development trajectories of the different projects, two approaches were observed in the study. We demarcate these approaches based on the way they conceptualised engagement. The most common approach – which we termed the ‘project-focused approach’ – observed in our findings was rather narrowly focused on project objectives. This approach is characterised by development of tools according to predetermined project guidelines with a focus on fulfilling the project milestones and deliverables according to the principles of good project governance. In the second approach – the so-called ‘user-focused approach’ – engagement with the relevant stakeholder group(s) is central to the development of the tool. The observed characteristics of both approaches are outlined in Table 3.

**Table 3.** The two approaches to developing engagement tools.

Phase	Project-focused approach	User-focused approach
Purpose	Narrow tool perception	Open tool perception
Prototyping	Moderated feedback	Open input and co-creation
Praxis	End-of-project dissemination	Ongoing mobilisation
Post-project continuity	Limited effort	Active search for alternative funding

Characteristics of the project-focused approach are visible in each distinct phase of the tool development process. In the early purpose-defining stage, projects applying this approach seem to already define the tool format and user benefit. As shown in the previous section, this could be a result of attempting to present the value of the tools that are to be developed to the stakeholders who will eventually use it and should be engaged in its development. As this approach seeks to clearly define the tool early in the project, the stakeholders are engaged on the basis of strictly defined guidelines and roles. The kinds of stakeholders engaged by such projects are often limited. Our results suggest that this may be driven by concerns regarding the feasibility of citizens' proposals and perceptions of their limited technical expertise. In the project-focused approach, the dissemination of the developed tools often takes place towards the end of the project. Our analysis indicates that most projects do not dedicate significant budgets to dissemination and largely rely on the existing channels of dissemination such as LinkedIn. With regard to post-project support and tool longevity, the project-focused approach involves following the obligatory guidelines imposed by funders, such as ensuring the project deliverables are available online for at least five years after the project ends. The results also show that some projects develop proposals for new projects by leveraging tools that have been particularly successful.

In our study, we also observe a distinctly different approach to tool development – the user-focused approach. In the early purpose-defining stages of projects, this approach entails involving various stakeholders, especially vulnerable or marginalised groups in conceptualising the tools. Projects employ monetary as well as non-monetary resources to ensure sufficient stakeholder participation. Further on, in the prototyping phase, this approach leaves ample space for an iterative process where user input is not limited to a single feedback session and may even allow for abandoning the initial tool formats and developing new formats that resonate with the received feedback. This is evident in the example we highlight on reworking a digital engagement tool developed for farmers within the AGROBRIDGES project. In the praxis phase, projects employing this approach implement communication plans and outreach strategies with the support of their local partners, based on trust-building and long-term approaches. However, the short project timelines that are characteristic of the Horizon Europe framework often prevent projects from fully realising this. As seen in the results section, interviewees frequently expressed their frustration with short timelines for implementing and disseminating their engagement tools and measuring the tools’ impact. Finally, when it comes to the post-project continuity, the user-focused approach was visible in the creative strategies applied to ensure sustained impact of the tools. These included identifying funding bodies to maintain and further improve the tools developed as a part of the project, as well as developing the tools into marketable solutions.

The two approaches represent distinct ways of developing engagement tools in European R&I projects. Each of these approaches presents a group of coherent characteristics. Namely, if a tool is narrowly specified in the first phase (project-focused approach), it will likely follow project-oriented approach characteristics such as limiting the feedback options. However, the results also point that these approaches are not always isolated from each other. Project managers may for example organise an inclusive tool co-creation session, while later in the project be restrained (regarding time, budget, and staff capacity) in how they approach dissemination. Therefore, the two approaches are distinct in their logics across development phases – with the project-focused approach prioritising predefined structures and objectives, and the user-focused approach emphasising adaptability and iterative development based on user input. However, this does not necessarily mean that the different approaches predetermine a development path of a tool.

### Transitioning towards socially robust engagement tools

The majority of the projects examined in this study applied the project-focused approach in their work, wherein tools are developed as deliverables within a predetermined project logic. This approach can be regarded as maintaining the status quo as it represents the dominant mode of operation. At the same time, some projects employ a user-focused approach in at least some aspects of tool development. In this approach, tools serve as a means to mobilise stakeholders around a specific topic, with an emphasis on their robustness. As demonstrated in the findings, such projects prioritise coordination strategies that include the early and continual involvement of users in tool conceptualisation.

However, even among projects that predominantly apply the project-focused approach, this choice is not only driven by a lack of interest in or knowledge about user engagement, the choice regarding the applied approach is also driven by structural constraints and requirements from the funder. The competitive nature of European R&I funding often pushes consortia to focus on project-focused aspects such as research excellence and deliverables in their project applications, rather than RRI aspects such as inclusion of vulnerable stakeholders, as they feel the former will be valued more highly ( Tabarés *et al.*, 2022). As a result, there is often insufficient time, funding, and institutional support to adopt a fully user-focused approach. Additionally, the tight timelines of EU projects, combined with the precarious employment conditions of many researchers and project staff, further reinforce a project-focused approach.

Since securing future funding is a constant concern, teams often prioritise meeting deliverables deadlines over engaging in iterative, user-focused tool development. This is compounded by the fact that the individuals who draft grant proposals are not always the same as those who later execute the projects. As a result, commitments to stakeholder engagement made at the proposal stage may prove unrealistic in practice, particularly when organisational resources are stretched thin. Projects might want to include stakeholders for various aims, including the normative belief that it is the *right thing to do* to include the users of a tool in its design, to the substantive belief that including perspectives of a range of stakeholders will increase the quality of the knowledge fed into developing the tools (these and other aims of stakeholder inclusion were recently summarised by Schmidt *et al.* (2020). Therefore, not incorporating stakeholder engagement in tool development could result in less robust tools.

**Figure 1.**
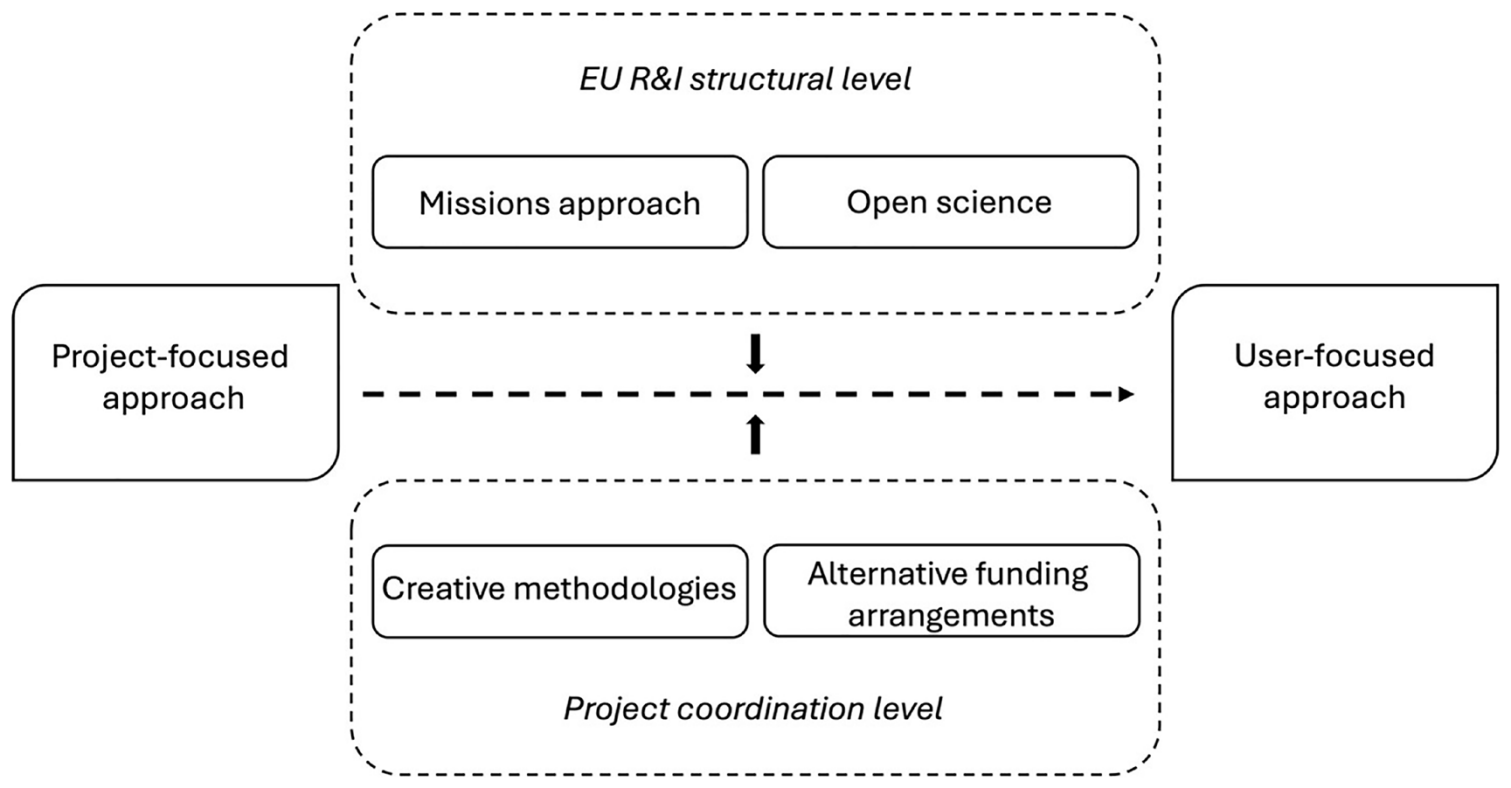
The transition to a user-focused approach to stakeholder engagement.

Despite these constraints, our findings indicate that there is a growing interest in working in a more user-focused way. While fully embedding this approach remains challenging, we observed instances where user-centredness was integrated into specific stages of tool development, often in an ad hoc manner or where project teams found ways to work around structural barriers. This suggests that there is momentum towards more engaged approaches, but for them to become the norm rather than the exception, changes are needed at multiple levels – ranging from how proposals are evaluated and funded to how projects are managed and supported throughout their lifecycle. Figure 1 presents a graphic overview of this momentum towards a user-focused approach and the changes needed to achieve it.

As shown in the figure, changes in both the EU R&I structural level as well as the project coordination are critical for moving towards a user-focused approach in developing and implementing stakeholder engagement tools. From our analysis, it is clear that solutions for creating more robust engagement tools come from both venues. At the same time, the finding that different stages of the same tool development can be dominated interchangeably by a project- and user- focused approach shows that these two approaches are not exclusive, but rather serve to explain the predominant rationales at the different stages of a project process. In that sense, few projects can be deemed exclusively project- or user- centered.

The growing interest in the user-focused approach is important as it emphasises the inclusion of diverse perspectives and knowledge sources to make tools more robust, while also considering the long-term viability of tools beyond a project’s lifespan. By prioritising broad stakeholder involvement, this approach aligns with the evolving European R&I landscape, which has shifted from a narrow focus on technological advancement to a more inclusive model of innovation that actively engages citizens. This transition, as outlined in the introduction, reflects broader trends in EU research policy, where engagement and co-creation have become key pillars of innovation. Furthermore, the user-focused approach resonates with the European Commission’s mission-oriented innovation principles, particularly those emphasised by Mazzucato (2018): broad participation, the co-production of robust knowledge, and interdisciplinary collaboration. However, despite policy-level commitments to RRI and Open Science, their implementation within EU-funded projects has been patchy ( Tabarés *et al.*, 2022). This may explain why the user-focused approach to tool development is emerging only in a partial or fragmented manner rather than as a widespread standard.

Nevertheless, the project-focused approach also has strengths that should not be overlooked. While it may be less oriented towards reflexivity and flexibility, it reinforces key governance principles essential for large-scale collaborative research. The structured nature of EU projects – defined by specific timelines, deliverables, and reporting procedures – ensures accountability, providing a clear framework for producing required knowledge and outputs within agreed parameters. These procedural elements, though sometimes perceived as restrictive, serve an important function in maintaining transparency and reliability within the research process. Moreover, what may appear as bureaucratic constraints within Horizon Europe could, in some cases, signal a need for greater skill development among project consortia. Conversely, if administrative requirements of project implementation take up capacities of project coordinators, there are less resources dedicated to ensuring the tools have genuine impact.

As Tenhunen-Lunkka and Honkanen (2024) suggest, the capacity to navigate project structures effectively – balancing compliance with flexibility – may be an area where additional training and institutional support could facilitate a more integrated approach. Rather than viewing engagement and structured project management as opposing forces, a more nuanced perspective might recognise the potential for their coexistence, leveraging the strengths of both approaches to enhance the impact and sustainability of R&I efforts.

### Limitations

While this study provides valuable insights into how tools for engagement reflect wider engagement efforts in European Research and Innovation processes, certain limitations should be highlighted regarding its reach. Firstly, due to the scope of the study, the focus was solely on the tool development process, without examining how different development approaches influence the usability of the tools. Future research could address this gap by investigating the practical implications of these approaches, as well as the long-term impact of engagement tools on stakeholder participation and decision-making in European R&I initiatives. Therefore, future research could look into whether user- and project-focused approaches result in substantially different tools (e.g. do tools developed by project-centered teams result in tools reproducing institutional logics?). On the same track, looking into how tool development approaches affect their conceptual RRI and Transformative basis could be a promising area of research. For example, while commercialisation approaches are broadly seen as worthwhile in ensuring post-project continuity, these types of strategies could limit the accessibility principles of RRI and thereby reduce the transformative potential of a tool.

Secondly, we recognise that this study has a specific thematic focus, with the studied projects mostly concerning agri-food themes, the bioeconomy and sustainable development. This is relevant as the agri-food system transformation efforts might entail specific types of participation as well as engagement institutionalised through living labs, food policy councils, and various collectives ( Garcia-Gonzalez & Eakin, 2019; Wiarda *et al.*, 2023). In order to ascertain whether the patterns identified through this study are visible in other areas of European R&I, research in additional fields of R&I should be further explored.

Additionally, the non-food specific projects that were analysed in this study did not diverge from the findings observed in the food-related projects. While this lack of apparent divergence is noteworthy, the emphasis on co-creation and sustainability central to both projects might make them more aligned with food system transformation efforts due to their higher participative potential, as explained earlier. To confirm this, further research is needed into the trends of engagement in R&I projects related to co-creation.

## Conclusion

In this study we explored how tools for stakeholder engagement are developed by Horizon Europe R&I projects. We did so by analysing 14 Horizon Europe projects and identifying their best practices as well as the challenges they faced in the creation of stakeholder engagement tools. These insights allowed for a development of wider approaches to development of tools for stakeholder engagement in European R&I projects. Illustrating how these approaches are implemented in the context of R&I projects allowed for broader findings on a mismatch of a rhetoric on open science practices and collaboration in EU R&I on the one hand, and the high administrative burden and inflexibility of the EU-level public funding on the other.

Using engagement tool development as a case highlighting the European R&I shift towards more RRI and Open Science principles helps underscore the multiscalarity of the issue (which has been discussed in the context of geographical perspective by Wiarda & Doorn, 2023) , with differing views on whether it should be addressed with the structural reform of the R&I system or project management reform. The case discussed in this study is also indicative of wider challenges the EU faces in engaging with the European public ( Gudek *et al.*, 2024). We specifically contribute to the literature by highlighting how a growing interest in a user-focused approach to stakeholder engagement in EU R&I projects is compatible with the wider structural shift towards RRI and Open science principles in Horizon Europe.

Additionally, while previous studies highlight the need for either a project management reform (for example Tenhunen-Lunkka & Honkanen, 2024), or stress the structural reproduction of injustice ( Luger *et al.*, 2023), this study indicates how both the project management and structural dynamics influence EU R&I implementation. It appears that the project-level as well as structural elements relate to the nature of how R&I is done, both supporting the project-focused approach and allowing for the emergence of a user-focused approach. Therefore, this study highlights how the elements of both the structural R&I level and the project coordination level both produce distinct approaches to implementing R&I projects in Europe. Finally, these considerations point to the importance of further exploring the dynamics of how the EU engages with citizens through R&I in the future to facilitate more responsive, robust, and accountable innovation.

### Ethical approval

Ethical approval was not required.

### Consent to participate

Written informed consent to record the interviews, analyse the resulting data, and publish the findings, including anonymised excerpts, was requested and obtained from all participants. Additionally, the rights of the participants were validated at the beginning of each interview, with the explicit right to retract their consent.

### Consent to publish

Written consent to publish the interview data, including anonymized text excerpts and generalised professional roles of the interview participants was gathered. The form of these data was briefly discussed at the beginning of each interview.

## Data Availability

The interview guide and the coding tree used in this study are hosted on the Open Science Framework database. The interview guide contains the overview of the themes and questions that were used to interview the study participants. DOI: 10.17605/OSF.IO/729DN [please cite: Gudek, L., Rao, M., & Broerse, J.E.W. (2025a) Stakeholder engagement in European research and innovation / Interview guide [dataset]. Open Science Framework. DOI 10.17605/OSF.IO/729DN] The coding tree contains an overview of the codes developed in the analysis process. DOI: 10.17605/OSF.IO/MZ4US [please cite: Gudek, L., Rao, M., & Broerse, J.E.W. (2025b) Stakeholder engagement in European research and innovation / Coding tree [dataset]. Open Science Framework. DOI 10.17605/OSF.IO/MZ4US] Both datasets are available under the CC-BY 4.0 licence and assigned unique DOI identifier. The datasets are listed in the reference list. Furthermore, the data used in the study concerns interview transcripts. While these have been anonymised, full transcripts might lead to projects, and consequently project staff to be identified. In addition, information shared by interviewees was at times sensitive and personal in nature and was shared with the authors under an understanding that it will not be further shared in its raw form. The corresponding author (Luka Gudek – l.gudek@vu.nl) can be contacted to receive parts of anonymised raw data upon reasonable request (from an email address associated with a research institution). Specifically, access to the data will be ensured for any persons interested in understanding the raw data from the transcripts, in order to replicate the research or conduct further studies. These raw data will include redactions of any personal information of the interviewees (including names, specific professional roles, project names, and organisation names).

## Footnotes

^1^ In this paper, Europe is referred to broadly as a geographical area, EU as the political community including all its legislative branches, and to the EC as the executive body managing the EU’s R&I policy.
