# Supplementary material for: Stakeholder engagement in European research and innovation: An investigation into how and why EU R&I projects develop engagement tools
Source: Open Res Eur. 2025 Sep 9;5:107. Originally published 2025 Apr 15. [Version 2] doi: 10.12688/openreseurope.19907.2 (PMC12138324; doi:10.12688/openreseurope.19907.2)
Supplement: Peer review history

# Reviewer report: Reviewer response for version 2

**DOI:** 10.21956/openreseurope.23013.r59775

**Published:** 2025-11-21

**Authors:** Johannes Starkbaum

**Affiliations:** 1 Institute for Advanced Studies (IHS), Vienna, Austria

I support indexing of this revised version

If applicable, is the statistical analysis and its interpretation appropriate?

Not applicable

Is the study design appropriate and is the work technically sound?

Yes

Is the work clearly and accurately presented and does it engage with the current literature?

Yes

Are the conclusions drawn adequately supported by the results?

Yes

Are sufficient details of methods and analysis provided to allow replication by others?

Partly

Are all the source data and materials underlying the results available?

Partly

Reviewer Expertise:

public engagement, open innovation, RRI, agriculture

I confirm that I have read this submission and believe that I have an appropriate level of expertise to confirm that it is of an acceptable scientific standard.

# Reviewer report: Reviewer response for version 2

**DOI:** 10.21956/openreseurope.23013.r59774

**Published:** 2025-11-21

**Authors:** Angelo Leogrande

**Affiliations:** 1 LUM University-Giuseppe Degennaro, Casamassima, Italy

The authors have answered the questions posed

If applicable, is the statistical analysis and its interpretation appropriate?

Yes

Is the study design appropriate and is the work technically sound?

Yes

Is the work clearly and accurately presented and does it engage with the current literature?

Yes

Are the conclusions drawn adequately supported by the results?

Yes

Are sufficient details of methods and analysis provided to allow replication by others?

Yes

Are all the source data and materials underlying the results available?

Yes

Reviewer Expertise:

Economics, finance, banking, research and development, machine learning, data analysis, data science.

I confirm that I have read this submission and believe that I have an appropriate level of expertise to confirm that it is of an acceptable scientific standard.

# Reviewer report: Reviewer response for version 2

**DOI:** 10.21956/openreseurope.23013.r59776

**Published:** 2025-09-23

**Authors:** Krzysztof Goniewicz

**Affiliations:** 1 Polish Air Force University, Dęblin, Poland

The authors have addressed all the requested revisions.

If applicable, is the statistical analysis and its interpretation appropriate?

Not applicable

Is the study design appropriate and is the work technically sound?

Yes

Is the work clearly and accurately presented and does it engage with the current literature?

Yes

Are the conclusions drawn adequately supported by the results?

Yes

Are sufficient details of methods and analysis provided to allow replication by others?

Yes

Are all the source data and materials underlying the results available?

Partly

Reviewer Expertise:

public health, disasters, emergencies

I confirm that I have read this submission and believe that I have an appropriate level of expertise to confirm that it is of an acceptable scientific standard.

# Reviewer report: Reviewer response for version 1

**DOI:** 10.21956/openreseurope.21534.r54068

**Published:** 2025-06-04

**Authors:** Angelo Leogrande

**Affiliations:** 1 LUM University-Giuseppe Degennaro, Casamassima, Italy

The article is well written, however it is possible to identify the following critical issues:

 • The article refers to the fact that a qualitative analysis was conducted through interviews. However, the essential characteristics of these interviews are not reported. Even if they are qualitative data, they can still be analyzed through classifications and the like.

 • The authors used the Atlas software to analyze the results of the qualitative analysis. However, it is not clear what type of qualitative methodology they used. Whether or not they used machine learning and in what way. Whether they simply limited themselves to a lexicometric analysis of the results obtained from the questionnaires. Or whether they translated the qualitative dimension into a quantitative dimension through the proposed software. It is necessary to better specify in the methodology what was achieved in terms of data analytics, data processing, data mining, data ingestion, data interpretation.

 • The authors refer to the fact that they used lines of code. It would be appropriate to make these lines of code public in an appendix specifically prepared at the end of the article, also to allow the possible replicability of the article's results.

 • The category of stakeholders and stakeholder management is often referred to. However, in the proposed literature there is no reference to Robert Edward Freeman who invented the very idea of ​​stakeholder theory. It would also be necessary to add a section to the article in which to delve deeper into the idea of ​​stakeholder management and highlight the possibilities of this idea - born in the corporate sphere - to serve the interests of public institutions and especially of the research and innovation policies of the European Union. In this sense, it would also be very useful to jointly analyze the idea of ​​stakeholder theory with the idea of ​​open innovation.

 • The introduction is too long and tends to also include the analysis of the literature. It would be appropriate to reorganize the article by dividing the introduction from the analysis of the literature. In the introduction, the research question should be expressed, the motivations that push this article to be original, any gap that one wants to fill in the studies on the topic. In the literature analysis, however, it is absolutely necessary to give space to the detailed treatment of references capable of bringing together the treatment of stakeholder theory in the public sphere in connection with the idea of ​​open innovation and innovation ecosystems inserted in the context of European public finance.

 • In table 1, it is necessary to add a column indicating the stakeholders most involved in the proposed research project. In fact, the article often speaks of stakeholders, but they are never defined in detail. Generally, each research project has its own definition of stakeholders. It would be very useful to define stakeholders also to highlight the fact that such projects can also partly differ in the sense of identifying the main stakeholders. For example, the article refers to citizens as stakeholders. Of course, in a broad sense, this may be true. However, when applying the stakeholder management model, we generally mean more limited categories of stakeholders that may concern, for example, company workers or consumers, or the various project partners.

 Following these changes, it is possible to orient the article towards publication.

If applicable, is the statistical analysis and its interpretation appropriate?

Yes

Is the study design appropriate and is the work technically sound?

Yes

Is the work clearly and accurately presented and does it engage with the current literature?

Yes

Are the conclusions drawn adequately supported by the results?

Yes

Are sufficient details of methods and analysis provided to allow replication by others?

Yes

Are all the source data and materials underlying the results available?

Yes

Reviewer Expertise:

Economics, finance, banking, research and development, machine learning, data analysis, data science.

I confirm that I have read this submission and believe that I have an appropriate level of expertise to confirm that it is of an acceptable scientific standard.

# Response

**Published:** 2025-08-26

**Authors:** Luka Gudek

**Affiliations:** Athena Institute, VU Amsterdam Athena Institute, Amsterdam, North Holland, The Netherlands

We would like to thank you for your constructive comments on our article. We have carefully assessed each of the points you raised, with special emphasis on the questions of methodology and data analysis, article structure, and theoretical perspectives on stakeholder engagement.

 Below, we provide a point by point response to each of your comments, along with an explanation of the corresponding change in the manuscript. Thank you again for your effort in the review process and we hope that these changes adequately address your concerns.

 On behalf of the co-authors,

 Luka Gudek --- Point by point responses: 1. Point on clarifying the use of the concepts of 'stakeholders' and 'stakeholder engagement' We rewrote a part of the theoretical section to refer more clearly to which specific strands of literature we draw from (literature on responsible research and innovation and challenge-led innovation). We now incorporate the role of Robert Edward Freeman in stakeholder engagement theory as well as Arlso Rip and Participatory Technology Development. Additionally, we specify stakeholder engagement within the paper to RRI and innovation literature where stakeholders are used to refer more broadly to societal actors like citizens, food producers, researchers, policy makers, and others. This approach fits the contribution of this paper more precisely, as with the paper we aim to contribute predominately with empirical knowledge on how stakeholder tools are developed in EU-led innovation processes. This is why we add additional theoretical grounding in these two branches, rather than making a connection to the business domain.

 "While engagement with various societal actors has become more prevalent in EU policy documents and academic literature, reflections on engagement are also explored in different scientific fields. The concept of stakeholder engagement gained salience in the 1980s in domains such as Business Organisation (cf. Freeman, 2010), Constructive Technology Assessment (Rip, 2018), and Participatory Technology Development (within agricultural development domain). More recently, in 2010s, the increased incorporation of engagement approaches into EU R&I has been a result of the Responsible Research and Innovation concept, which builds on previous research on stakeholder engagement, emphasising inclusion, anticipation, responsiveness and reflexivity in innovation processes (Burget et al., 2017).

 Within the European R&I projects, the institutionalisation of RRI took place through the development of methods to engage with the public, as well as making those freely available ( Owen et al., 2021). Within RRI, those to be engaged are often referred to as stakeholders (cf. Koch, 2020) to encompass the different groups and interests to be engaged. As the work on engaging various societal actors in policy grew in importance, it was also integrated into research within the sustainability transitions field, with engagement of stakeholders being emphasised as crucial for achievement of transformative objectives (Huttunen et al., 2022)." 2. Point on restructuring the introduction section We restructured the introduction to clearly state the research question and while we refer to the existing literature, we do so to highlight the gaps in existing research (e.g. lack of empirical knowledge on participation in RRI and transformative innovation processes, as well as a lack of implementation knowledge on engagement in European R&I). For example: "At the same time, research engaging with RRI tools points to a disparity between theory and practice, as well as a need for more empirical knowledge on implementation of RRI processes (Marschalek et al., 2017)."

 In the theory section, we significantly expand on the literature on European transformative innovation, stakeholder engagement (as well as clarifying we draw on sustainability transitions and transformative innovation fields' conceptions of engagement). We elaborate on these in other comments.

 Finally, a research question has now been explicitly added: "To achieve that, the study tackles the question: How are engagement tools developed in R&I projects and what are the underlying logics of these processes?"

 The length of the introduction was kept within the average of Open Research Europe publishing (500-800 words). 

 3. Point on clarifying the empirical use of stakeholders and defining the tool target groups in Table 1 A short description on who the targeted stakeholder are was added for each project in Table 1 to clarify who the stakeholders are.

 Additionally, while we refer to stakeholders, we do not apply the stakeholder management model, but rather draw on the theory on RRI and challenge-led innovation when discussing stakeholder engagement. We have now more explicitly outlined which literature we do and do not draw from in the theory section. We explain this in more detail on the comment on stakeholders and Robert Edward Freeman.

 Furthermore, we outlined how we engage with the term stakeholder in the study itself, in the methods section.

 "Table 1 presents an overview of the included projects, and the tools developed by them, as well as which stakeholders they tried to engage. As the projects attempt to engage diverse societal actors, such as policy makers, food producers, researchers, businesses, we refer to these collectively as stakeholders taking part in stakeholder engagement processes. When appropriate, we refer to specific stakeholder groups." 4. The point on clarifying interview data The code tree and interview guide have now been included as appendices to the paper, increasing transparency and allowing for greater replicability. 5. Point on clarifying the data analysis and coding process We would like to clarify that the study employed a qualitative thematic analysis methodology, drawing on Saldaña’s (2021) framework for manual coding. The term 'code' in this context refers to analytical labels assigned to excerpts of interview transcripts during the process of qualitative data interpretation, not lines of programming code. Atlas.ti was used solely as a tool to support this manual process, by allowing us to organise, retrieve, and compare segments of text based on the assigned codes. No machine learning techniques, lexicometric analysis, or quantitative transformation of the data were used. The entire coding and interpretation process was conducted by the research team, based on close reading and constant comparison of the transcripts.

 In response to your suggestion regarding transparency and replicability, we have now included the full coding tree used in our analysis as an appendix. This provides a complete overview of the codes developed during our thematic analysis. We hope this clarifies the nature of our methodological approach.

# Reviewer report: Reviewer response for version 1

**DOI:** 10.21956/openreseurope.21534.r54296

**Published:** 2025-06-02

**Authors:** Krzysztof Goniewicz

**Affiliations:** 1 Polish Air Force University, Dęblin, Poland

Your article presents a rich and well-structured qualitative investigation into how EU R&I projects develop stakeholder engagement tools. I found the phased model (Purpose, Prototyping, Praxis, Post-project continuity) and the typology of project-focused vs. user-focused approaches both original and valuable. At the same time, I have a few questions and suggestions that may help sharpen your analysis and strengthen your conclusions:

 Can you clarify why the study focuses primarily on agri-food and bioeconomy projects? I understand the relevance of Food2030, but how do you see your findings applying to other sectors of Horizon Europe?

 Could some of your conclusions be sector-specific?

 You briefly mention generalisability in the limitations, but I suggest introducing this question earlier, perhaps in the Methods or Discussion — to help readers frame the scope.

 The distinction between project-focused and user-focused trajectories is a useful analytical lens. At the same time, I find the boundary between these categories occasionally too rigid. Several examples in your data suggest hybrid practices.

 Could you expand your framework to reflect a spectrum or continuum rather than a binary? Would a typology with intermediary cases  better reflect the realities of Horizon Europe project dynamics?

 You refer to theoretical sampling and mention saturation after 10–11 interviews. Can you explain how saturation was assessed across such a diverse sample? Did you observe differences in tool development logics between bioeconomy and agri-food projects? Between digital tools and analogue ones?

 A brief matrix or summary of project types, tools, and their assigned approach would enhance transparency.

 You raise important issues about whose voices shape engagement tools. I’d like to see this developed further. When certain groups are excluded from co-creation, what are the consequences? Do tools risk reproducing institutional logics rather than transforming them?

 Can you engage more with literature on co-optation and participation fatigue?

 The analysis of post-project challenges is one of the strongest sections. You highlight multiple strategies for sustaining tools, but I wonder: Which ones have actually worked? Are there examples where uptake by municipalities, ministries, or commercial partners has occurred?

 Could you reflect more critically on the tension between open science ideals and commercialisation logic? When a tool becomes a product, how does this affect inclusivity and access?

 Given the qualitative nature of the work, I understand the restrictions on data sharing. Still, would you consider including your full coding tree, code definitions, or a sample coded excerpt as supplementary material?

**Minor points**

- The Abstract could specify the number of projects and interviews for clarity.
- A schematic diagram illustrating the two approaches across the four phases might make your conceptual contribution more accessible.
- In the Discussion, when referring to Horizon Europe’s administrative burdens, can you be more specific? Are there procedural or reporting rules that especially limit co-creative practices?

**Conclusion**

 This is a timely and methodologically sound paper that contributes new insight to the literature on stakeholder engagement in EU R&I. Addressing the questions above could help refine your conceptual model, clarify your empirical base, and enhance the practical relevance of your findings for project coordinators, funders, and policymakers.

If applicable, is the statistical analysis and its interpretation appropriate?

Not applicable

Is the study design appropriate and is the work technically sound?

Yes

Is the work clearly and accurately presented and does it engage with the current literature?

Yes

Are the conclusions drawn adequately supported by the results?

Yes

Are sufficient details of methods and analysis provided to allow replication by others?

Yes

Are all the source data and materials underlying the results available?

Partly

Reviewer Expertise:

public health, disasters, emergencies

I confirm that I have read this submission and believe that I have an appropriate level of expertise to confirm that it is of an acceptable scientific standard, however I have significant reservations, as outlined above.

# Response

**Published:** 2025-08-26

**Authors:** Luka Gudek

**Affiliations:** Athena Institute, VU Amsterdam Athena Institute, Amsterdam, North Holland, The Netherlands

We would like to thank you for your constructive comments on our article. We were happy to read your positive response on the relevance of the phased model and the project-user focused typology.

 We have carefully assessed each of the points you raised, with special emphasis on the questions of context of agri-food domain and generalisability potential, the typology, methodological considerations and data sharing, as well as engagement-related tensions in tool tool development stages.

 Below, we provide a point by point response to each of your comments, along with an explanation of the corresponding change in the manuscript. Thank you again for your effort in the review process and we hope that these changes adequately address your concerns.

 On behalf of the co-authors,

 Luka Gudek --- Point by point responses: 1. Point on tensions, risks, and consequences of stakeholder engagement We incorporate perspectives on failures and risks of participation specifically in RRI and Mission oriented innovation - these highlight the political resistance and tensions that surface in innovation processes addressing societal issues; even when the participation processes are organised well. Furthermore, we highlight stakeholder fatigue and how the processes of participation can reduce trust of stakeholders when mismanaged.

 "Failing to meaningfully engage with actors in these processes can have consequences such as stakeholder fatigue when stakeholders are engaged in extractive ways rather than seeking their input at all stages of projects (Bracken et al., 2015). Additionally, empirical research shows that bureaucratic and political resistance to participative processes regularly means that engagement does no’t translate to policy action (Wesselink et al., 2011). In R&I processes, the input of stakeholders is often not evaluated equally, with citizens’ voices frequently being undervalued. Furthermore, even when meaningful participation processes are designed, the political nature of missions might reinforce tensions and polarisation around certain societal issues (Janssen et al., 2021)."

 Additionally, we open up the question of whether the user- and project- centered approaches also correspond to substantially distinct project outputs - e.g. do project-centered approaches result in tools that reproduce institutional logics. 2. Point on clarifying the agri-food domain focus The focus on food systems related project is a result of all authors working on the CLEVERFOOD project at the time of the project. CLEVERFOOD creates networks of projects targeting food systems within Horizon Europe. We explain this in the manuscript as shown below. The question on sectoral limits and generalisability is answered in the subsequent review point.

 "The study focuses on a subset of Horizon Europe projects, namely the projects under the Food 2030 umbrella, attempting to address the challenges of food system transformation. Potential projects were identified via a desk study involving the analysis of all ongoing and recently concluded Horizon Europe food and agriculture projects. This thematic subset of projects is a result of the authors’ work on the CLEVERFOOD project which engages broadly with food system related projects." 3. Point on potential sector-specificity of the conclusions Indeed, some of the conclusions may be sector specific, which we refer to in the study limitations, with the indication of some early research on the engagement emphasis in food missions. The text is shown below.

 "Secondly, we recognise that this study has a specific thematic focus, with the studied projects mostly concerning agri-food themes, the bioeconomy and sustainable development. This is relevant as the agri-food system transformation efforts might entail specific types of participation as well as engagement institutionalised through living labs, food policy councils, and various collectives ( Garcia-Gonzalez & Eakin, 2019; Wiarda et al., 2023)." 4. Point on incorporating a reflection on limitations in the methodology We added new text explaining the potentially limited scope of the study in the methodology section in order to provide the reader some context before the results. The text is shown below:

 "Finally, none of the interviewees pointed to contrary experiences in non-food projects they had coordinated. Nevertheless, the strong representation of agri-food projects suggests that the findings might not be fully generalisable to all thematic areas of Horizon Europe R&I landscape and may need to be further validated." 5. Point on the potential rigidity of the framework of user- and project-centered approaches We agree that the project- and user- centered approaches should not be seen as binary or exclusive. Therefore, we add a clarification to the discussion expanding on the existing examples, mentioning that the two approaches can help explain the different rationales at specific stages of tool development, but projects on a whole are rarely exclusively project- or user- centered.

 Where we see a strength of the current figure is in presenting the factors affecting the overall approach to tool development (EU R&I structural level and the Project coordination level) and the overall shift towards the user-focused approach. However, in the revised version of the manuscript, we explain in more detail about the spectrum of approaches to tool development.

 "At the same time, the finding that different stages of the same tool development can be dominated interchangeably by a project- and user- focused approach shows that these two approaches are not exclusive, but rather serve to explain the predominant rationales at the different stages of a project process. In that sense, few projects can be deemed exclusively project- or user- centered." 6. Point on tensions between open science and commercialisation principles We expand on this in the revised version of the manuscript by adding a reflection on how questions of tensions in tool development approaches can result in unexpected consequences for the underlying RRI and TIP principles of tool development. We do this in the discussion and propose future research to resolve the issue.

 "Therefore, future research could look into whether user- and project-focused approaches result in substantially different tools (e.g. do tools developed by project-centered teams result in tools reproducing institutional logics?). On the same track, looking into how tool development approaches affect their conceptual RRI and Transformative basis could be a promising area of research. For example, while commercialisation approaches are broadly seen as worthwhile in ensuring post-project continuity, these types of strategies could limit the accessibility principles of RRI and thereby reduce the transformative potential of a tool." 7. Point on specifying the number of interviews in the abstract The 'methods' subsection of the abstract now mentions the number of interviews as follows: "This study adopts a qualitative approach, with conducting semi-structured interviews with 22 participants representing 14 Horizon Europe projects on topics of agri-food, bioeconomy, and sustainability." 8. Point on specifying the administrative burdens of Horizon Europe project implementation We delve into the issue of how these administrative burdens manifest themselves in more detail in the section Transitioning towards socially robust engagement tools:

 "The competitive nature of European R&I funding often pushes consortia to focus on project-focused aspects such as research excellence and deliverables in their project applications, rather than RRI aspects such as inclusion of vulnerable stakeholders, as they feel the former will be valued more highly ( Tabarés et al., 2022). As a result, there is often insufficient time, funding, and institutional support to adopt a fully user-focused approach. Additionally, the tight timelines of EU projects, combined with the precarious employment conditions of many researchers and project staff, further reinforce a project-focused approach.

 Since securing future funding is a constant concern, teams often prioritise meeting deliverables deadlines over engaging in iterative, user-focused tool development. This is compounded by the fact that the individuals who draft grant proposals are not always the same as those who later execute the projects. As a result, commitments to stakeholder engagement made at the proposal stage may prove unrealistic in practice, particularly when organisational resources are stretched thin." 9. Point on data saturation and differences in specific properties of specific tools and projects We agree that our sample included a range of projects across thematic domains and tool formats. However, saturation in this study was assessed specifically in relation to recurring patterns in the structure of the tool development process, participants’ motivations to develop tools, and the challenges and adaptations encountered across development phases. These were the central themes guiding our analysis, and it was on these dimensions that we observed sufficient thematic repetition across interviews.

 While there were differences across projects in terms of their goals, outputs, and target user groups, we did not find meaningful differences in how participants described the phased logic of tool development. Both agri-food and bioeconomy projects, for example, followed a similar narrative arc when discussing how engagement tools were conceptualised, prototyped, implemented, and sustained. For this reason, we chose not to add further distinctions (e.g. sector or tool type) to Table 1, as it is intended to provide a descriptive overview rather than support a comparative analysis. We have now clarified this rationale in the revised methods section as follows:

 "As our analytical focus was on recurring patterns in how the tool development process was structured, participants’ motivations, and responses to key challenges, saturation was assessed in relation to these themes rather than across sectoral or technological dimensions." 10. Point on specifying the findings in the post-project stage We appreciate the positive feedback regarding the analysis of post-project challenges. In response to your suggestion, we have revised the relevant section of the manuscript to include concrete examples from our data. These include projects that leveraged their tools as the basis for new project proposals (e.g., Fit4Food leading to CLEVERFOOD, AgroBridges drafting a follow-up proposal), as well as tools adopted or further explored by NGOs, municipalities, or institutional actors (e.g., FOX, ECO-READY).

 Regarding the third valorisation pathway i.e., commercialisation, although some interviewees shared that their teams were exploring this pathway, these efforts were at a preliminary or confidential stage at the time of the interviews. To respect participant confidentiality and avoid reporting outcomes not yet realised, we have chosen not to include specific examples of commercialisation in the manuscript.

 The aforementioned changes are reflected in the manuscript throught the addition of the following text:

 "For example, the CLEVERFOOD project originated from the earlier FIT4FOOD project, serving as a continuation mechanism to expand and consolidate engagement tools. In the case of AgroBridges, the consortium was actively developing a new project proposal to build upon the tools they had created. The FER-PLAY team also reported that they were exploring opportunities to capitalize on the outcomes of their project by applying for new funding that would allow continued development of their tools."

 "In the FOX project for instance, a regional partner engaged with their local municipality to explore the possibility of continuing the use of a mobile unit and related app created as part of the project. Similarly, in ECO-READY, the project team discussed the possibility of leveraging strategic partnerships, including with the European Commission’s Joint Research Centre, to embed their tools into external platforms, while also using Living Labs to explore future sustainability options." 11. Point on sharing the coding information The code tree and interview guide have now been included as appendices to the paper, increasing transparency and allowing for greater replicability. 12. Point on including a schematic of the approaches and phases Thank you for this thoughtful suggestion. We agree that visual clarity is important, and we carefully considered including a schematic diagram. However, given that Table 2 outlines the four development phases and Table 3 maps the project-focused and user-focused approaches across those phases, we felt that the conceptual structure was already effectively communicated. To avoid redundancy and maintain a clean layout, we have decided not to include an additional schematic at this time.

 However, we have added additional information qualifying the existing framework, adding additional explanation on its applicability (non-exlusivity of the two approaches, how they may differ across stages, and which stakeholders these processes include).

# Reviewer report: Reviewer response for version 1

**DOI:** 10.21956/openreseurope.21534.r54067

**Published:** 2025-05-28

**Authors:** Johannes Starkbaum

**Affiliations:** 1 Institute for Advanced Studies (IHS), Vienna, Austria

The article by Gudek et al. explores how tools supporting stakeholder engagement are developed, deployed, and sustained within EU R&I projects. It critically engages with the practical implementation of these and how they relate to the broader R&I structures. Some points to consider:

- ***Critical perspective with EU R&I (introduction)*** *: While the article shortly addresses the “projectified” nature of European R&I in the introduction, I miss a more critical perspective on EU ambitions for stakeholder engagement. There exist many publications that engage critically e.g. with RRI, its conceptualization, implementation etc. The same goes for Mission Orientation. The article would benefit from integrating some reflection on the R&I policy level at this stage, especially as you later engage in criticism of how R&I is organized on a broader scale (discussion). *
- ***Definition of tools (introduction)*** *: The definition of engagement tools in the introduction is quite vague, referring to “actionable guidelines and practical processes”. This makes sense given the empirical span of aspects/tools covered in this study, but yet it would be good to give some concrete examples and in which phases of projects they are planned and employed. *
- ***Concrete examples (results)*** *: In line with the point above, it would help the reader to provide more concrete examples of engagement tools in the results section. Often, it is unclear if arguments mainly refer to things like workshops, policy briefs or art interventions, which makes a difference. *
- ***Interview questions (methods section)*** *: Some information on the rough topics of the interview questions would help to make data collection more transparent*
- ***Coding (methods section)*** *: You depart from grounded theory and the article of Saldaña 2021 to describe your open coding. Did you stop at this stage or have further steps of integration, like axial or selective coding, been employed? Consider adding some more information about how you developed a red threat or categories, i.e. broader topics emerging from single codes. *
- ***Timeframe (introduction, results)*** *: You speak of “tools developed and deployed within these projects” (p.4). But aren’t many activities you refer to in proposal phases not the project as such? One could argue that the proposal is part of the project, but it might be good to clarify. This is something which is also relevant for the results section, e.g. purpose section (which might mainly refer to proposal phase?). *
- ***Project-focused and user-focused approach (discussion*** *): The use of these ideal typical types of practices for tool development and application are interesting and cover an important aspect to consider for the topic of the study *

***Minor issues: ***

- *P2: You write that “This study contributes to the broader discourse on sustainability innovation…”. To me, it is not clear why the term sustainability is prominently referred to in this context as I don’t see this as a main ambition of this article. *
- *P4: “…ranging from food security and the climate crisis to maintaining global competitiveness” reads as if the Eu would tackle these only to maintain competitiveness, which is a key aspect but not the only driver. *

***Disclosure of (potential) conflicts of interest***

*More than ten years ago, I worked with one of the authors on a research project but since then we haven’t had any contact. I see no conflict of interest for this review.*

If applicable, is the statistical analysis and its interpretation appropriate?

Not applicable

Is the study design appropriate and is the work technically sound?

Yes

Is the work clearly and accurately presented and does it engage with the current literature?

Yes

Are the conclusions drawn adequately supported by the results?

Yes

Are sufficient details of methods and analysis provided to allow replication by others?

Partly

Are all the source data and materials underlying the results available?

Partly

Reviewer Expertise:

public engagement, RRI, agriculture

I confirm that I have read this submission and believe that I have an appropriate level of expertise to confirm that it is of an acceptable scientific standard.

# Response

**Published:** 2025-08-26

**Authors:** Luka Gudek

**Affiliations:** Athena Institute, VU Amsterdam Athena Institute, Amsterdam, North Holland, The Netherlands

We would like to thank you for your constructive comments on our article. We have carefully assessed each of the points you raised, with special emphasis on the questions of the critical perspectives on EU R&I, definitions and concrete examples of tools, as well as the methodological aspects of the study.  Below, we provide a point by point response to each of your comments, along with an explanation of the corresponding change in the manuscript. Thank you again for your effort in the review process and we hope that these changes adequately address your concerns. On behalf of the co-authors,

 Luka Gudek Point by point responses: 1. Point on a more critical reflection on EU RRI and Missions In the revised version of the article, we add a reflection in the theory section highlighting the critical literature (RRI and MOIP) on the challenges of engagement led by these concepts as well as a short reflection within the EU context. For example: "Furthermore, more critical perspectives on engagement in transformative R&I point out that mission-led approaches sometimes fail to resolve issues such as stakeholder monotony (cf. Kirchherr et al., 2023), and lack of consideration for geographical diversity (Uyarra et al., 2025)."

 We return to this in the discussion by referring to the challenges of engagement in supranational transformative innovation. 2. Point on clarifying how the 'Purpose' stage fits in the project timeline We see the Purpose stage encompassing activities largely within the project timeline. While the very general ideas of tools can be outlined in the project proposals (e.g. "development of a digital engagement platform for farmers"), this kind of a description still needs to be operationalised through a long process. This process usually includes understanding the needs that the tool should fulfil, the feasible format and functionalities, as well as the content. This is clarified in the text as shown below:

 "While a general idea of a tool might be developed before the project as a part of the proposal, the critical conceptualisation of the tool happens at the beginning of the project." 3. Point on clarifying how the concept of 'tools' is used Following your suggestion, we have added some examples of tools to make the concept of engagement tools more understandable for the reader:

 "Practically, these tools can take shape as scripts for multi-stakeholder workshops, digital data sharing platforms, or networking apps." 4. Point on incorporating more examples of tools We have now adjusted the results at several points to make more explicit references to the types of tools being discussed:

 On the other hand, user groups such as citizens were perceived as less knowledgeable, particularly about the feasibility of implementing certain changes "ideas for technical tools like apps and websites that facilitated collaboration."

 "When it came to developing tools based on digital platforms," Partners with IT expertise such as coders and web developers often had different views on the feasibility of implementing user feedback compared to project coordinators, or research partners, often leading to discord.

 Once the translation was underway or completed, making adjustments to the tools "(whether session outlines, or digital platforms)" also became a major challenge.

 For "digital tools", that required users to create accounts, it was possible to collect additional data about tool use.

 "While digital tools like apps and websites are vulnerable to becoming unusable due to a lack of maintenance, even methodologies and session outlines for workshops can become inaccessible if their hosting expires or external links in PDFs break over time." 5. Point on referring to *sustainability* innovation The sustainability-focused innovation references the policy grounding of the projects that are the empirical basis of the article. These projects are based in the European Green Deal and Food2030 frameworks that emphasise sustainability aspects of innovation.

 We realise that within the abstract the reference to sustainability might cause confusion so we removed it and refer simply to "innovation". Additionally, we expanded on the introduction to more clearly reflect the gap that the study addresses. More specific overview of this is available in other comments. 6. Point on sentence clarity We have now adjusted the wording to be more straight forward: "In recent decades, Europe has grappled with several challenges, including geopolitical crises, the climate crisis and biodiversity loss, maintaining global competitiveness, and others." 7. Point on interview questions We now include the interview guide as an appendix to improve the transparency of our data collection process. 8. Point on additional information in the coding process In the subsection 'data analysis' it is now explained that we undertook a second coding step following Saldana's axial coding process:

 "Subsequent to open coding, we engaged in a second cycle of analysis to group these codes into broader conceptual categories. Drawing on principles of axial coding (Saldaña, 2021), we examined relationships among the codes, explored underlying patterns, and grouped them into higher-order categories. Through this process, it became evident that participants consistently described their activities in chronological sequences, structuring their accounts around distinct stages of tool development and dissemination.Based on this, we identified four phases of the tool development and dissemination process, as described in the consequent section of this paper. The 46 codes that were created during the previous analysis step were then assigned to one or more of the stages." 9. Point on methodological transparency for potential replication The code tree and interview guide have now been included as appendices to the paper, increasing transparency and allowing for greater replicability.
